# Comparative Assessment of CMR-Determined Extracellular Volume Metrics in Predicting Adverse Outcomes

**DOI:** 10.3390/jcm14020382

**Published:** 2025-01-09

**Authors:** Katharina Mascherbauer, Christina Kronberger, Carolina Donà, Matthias Koschutnik, Varius Dannenberg, Michael Poledniczek, Laura Lunzer, Christian Nitsche, Franz Duca, Gregor Heitzinger, Kseniya Halavina, Dietrich Beitzke, Christian Loewe, Elisabeth Waldmann, Philipp E. Bartko, Julia Mascherbauer, Christian Hengstenberg, Andreas A. Kammerlander

**Affiliations:** 1 Division of Cardiology, Medical University of Vienna, Waehringer Guertel 18-20, 1090 Vienna, Austria; katharina.mascherbauer@meduniwien.ac.at (K.M.); christina.kronberger@meduniwien.ac.at (C.K.); carolina.dona@meduniwien.ac.at (C.D.); matthias.koschutnik@meduniwien.ac.at (M.K.); varius.dannenberg@meduniwien.ac.at (V.D.); laura.lunzer@gesundheitsverbund.at (L.L.); christian.nitsche@meduniwien.ac.at (C.N.); franz.duca@meduniwien.ac.at (F.D.); gregor.heitzinger@meduniwien.ac.at (G.H.); kseniya.halavina@meduniwien.ac.at (K.H.); philippemanuel.bartko@meduniwien.ac.at (P.E.B.); christian.hengstenberg@meduniwien.ac.at (C.H.); 2 Division of Cardiovascular and Interventional Radiology, Medical University of Vienna, Waehringer Guertel 18-20, 1090 Vienna, Austriachristian.loewe@meduniwien.ac.at (C.L.); 3 Division of Gastroenterology and Hepatology, Medical University of Vienna, Waehringer Guertel 18-20, 1090 Vienna, Austria; elisabeth.waldmann@meduniwien.ac.at; 4 Department of Internal Medicine 3, Karl Landsteiner University of Health Sciences, University Hospital St. Pölten, 3100 St. Pölten, Austria

**Keywords:** extracellular volume metrics, CMR, cardiac T1 mapping, iECV

## Abstract

**Background:** Extracellular volume (ECV) by cardiovascular magnetic resonance (CMR) imaging is associated with disease burden and clinical outcomes. Recent studies in patients with valvular heart disease (VHD) have suggested that the indexed total ECV (iECV) = ECVx(LV_mass_/1.05)/body surface area may supersede ECV in terms of prognostication. In this study, we aimed to compare the prognostic capability of conventional ECV and iECV in an all-comer CMR cohort. **Methods:** From January 2012 to 2023, ECV and iECV were measured in consecutive CMR patients. Adverse outcomes were defined as a composite of hospitalization for heart failure (HF) and/or death. All patients underwent transthoracic echocardiography within 3 weeks of CMR. **Results:** Overall, 1525 patients (44% female, mean age 65 ± 18 years) were included. The mean ECV was 29 ± 9% and the mean iECV was 21 ± 13 mL/m^2^. During 52 ± 36 months of follow-up, 414 (27%) events occurred. Both ECV (HR = 1.04, 95% CI = 1.04–1.05, *p* < 0.001) and iECV (HR = 1.03, 95% CI = 1.02–1.03, *p* < 0.001) were significantly associated with outcomes. Having been stratified for ECV and iECV tertiles, Kaplan-Meier analyses showed a significant association with event-free survival for both parameters (log-rank, *p* < 0.001 for both; central illustration). Regarding multivariate analysis, adjusted for age, sex, left ventricular function, and NT-proBNP, both ECV and iECV remained independently associated with the composite endpoint (ECV: HR = 1.31, 95% CI = 1.20–1.44, *p* < 0.001; iECV: HR = 1.17, 95% CI = 1.06–1.29, *p* = 0.002). In addition, ECV was significantly associated with aortic valve velocity (*p* < 0.001) pertaining to echocardiography, whereas iECV did not show an association (*p* = 0.41). **Conclusions:** Both conventional ECV and iECV provided profound prognostic information regarding the risk of HF hospitalizations and death. However, iECV, which is more complex to determine, did not add value.

## 1. Background

Parametric mapping techniques in cardiac magnetic resonance (CMR) imaging are considered state-of-the-art and indispensable for myocardial tissue characterization. Several metrics, including native T1-mapping and extracellular volume fraction (ECV), have been validated in numerous preclinical and clinical settings [1,2,3,4]. ECV represents the myocardial extracellular volume fraction.

Recently, the “total fibrosis burden of the heart” was introduced as a novel parameter, referred to as indexed ECV (iECV). iECV incorporates left ventricular (LV) mass and body surface area (BSA) and is calculated as:

iECV = ECV × (LV_Mass_/1.05)/BSA

where 1.05 g/mL is defined as the specific gravity of the myocardium [5].

In specific populations, such as patients with aortic valve disease, the prognostic value of iECV has been reported to be superior to traditional ECV [6,7]. In aortic stenosis (AS), myocardial fibrosis has been established as a key pathological process that drives the transition from hypertrophy to heart failure (HF). Subsequent research has demonstrated that in patients with valvular heart disease (VHD), including both AS, aortic regurgitation (AR), and mitral regurgitation, myocardial fibrosis, as quantified by iECV, was correlated with clinical outcomes following VHD intervention [6,8]. Based on these results, iECV was discussed as a tool for preoperative risk assessment [6,7,8,9]. However, apart from these few investigations, the evidence supporting the potential superiority of iECV over conventional ECV for the prognostication of patients with valvular and non-VHD is sparse. In this large all-comer CMR cohort, we aimed to compare the prognostic power of iECV with conventional ECV in consecutive patients with various cardiovascular diseases (CVD).

## 2. Methods

### 2.1. Study Design

This post hoc analysis of a prospective registry study included consecutive all-comers for CMR between January 2013 and January 2023. Within 3 weeks of CMR, the patients underwent a comprehensive echocardiographic assessment. The study was conducted at the Vienna General Hospital, a university-affiliated tertiary care center with a high-volume multimodality imaging facility. All participants gave written informed consent, and the Institutional Review Board of the Ethik Commission of the Medical University of Vienna approved the study protocol (EK #2036/2015, approval date: 15 December 2015).

The primary endpoint was defined as a composite of HF hospitalization and all-cause death. We retrieved the dates and causes of death from in-hospital charts, the national death registry, and nationwide medical records. Two study team members served as an adjudication committee for each event and were blinded to CMR data.

### 2.2. Patient Population

At the time of CMR, demographic data (age, sex, body mass index [BMI], BSA) and comorbidities were assessed. These included hypertension (≥140/90 mmHg or antihypertensive treatment), atrial fibrillation (present at the time of CMR or documented in the medical history), diabetes (fasting blood glucose level > 126 mg/dL, HbA1c ≥ 6.5%, or use of anti-diabetic medication), hyperlipidemia (total serum cholesterol 240 mg/dL or use of cholesterol-lowering medication), chronic obstructive pulmonary disease (documented in the medical history), peripheral artery disease (reported in the medical history), chronic kidney disease (eGFR < 40 mL/min/1.73 m^2^, MDRD formula), and previous myocardial infarction, which was defined both by medical history and CMR. In addition, blood tests, including the measurement of cardiac enzymes (including NT-pro BNP), were performed before the CMR scan.

### 2.3. Cardiac Magnetic Resonance Imaging

All patients underwent the CMR examination on a 1.5-T scanner (Avanto Fit, Siemens Healthineers, Erlangen, Germany) following standard protocols that included LGE imaging [0.15 mmol/kg gadobutrol (Gadovist; Bayer Vital GmbH, Leverkusen, Germany)] if the estimated glomerular filtration rate was >30 mL/min/1.73 m^2^. At the time of insertion of the intravenous cannula, blood was drawn for hematocrit and serum creatinine measurement. A semiautomatic approach was used to determine LGE and other measurements, applying dedicated software (cmr42, Circle Cardiovascular Imaging Inc., Calgary, AB, Canada). A threshold of five standard deviations (SD) above the mean signal intensity of the reference myocardium was defined.

Electrocardiographically triggered Modified Look-Locker Inversion Recovery (MOLLI) using Myomaps^®^ was used to generate an inline, pixel-based T1 map by acquiring images over several heartbeats with shifted T1 time, inline motion correction, and inline calculation of the T1 relaxation curve within one breath hold. T1 sequence parameters were as follows: a starting inversion time of 120 ms, an inversion time increment of 80 ms, a reconstructed matrix size of 256 × 218, and a measured matrix size of 256 × 144 (with a phase-encoding resolution of 66% and a phase-encoding field of view of 85%). T1 maps were created before and 15 min after the contrast agent application.

Extracellular volume fraction measurements were derived from a previously described formula using pre- and post-contrast myocardium and blood pool T1 times and hematocrit [10]; they were expressed as percentages. Representing the absolute amount of myocardial extracellular space, iECV was calculated by multiplying global ECV by the indexed LV end-diastolic myocardial volume; it was expressed in milliliters per meter squared (mL/m^2^) [6,9].

### 2.4. Echocardiography

All patients underwent a standard echocardiogram by board-certified cardiologists using commercially available equipment (Vivid E95, GE Healthcare, Acuson Sequoia, Siemens, München, Germany). Following current recommendations, cardiac morphology was examined in standard 4- and 2-chamber views [11]. AS was quantified using an integrated approach as described in respective guideline recommendations. Low-flow, low-gradient AS was defined as an aortic valve velocity under 4 m/s and a LV stroke volume < 35 mL/m^2^ [12].

### 2.5. Statistical Analysis

All statistical analyses were performed using Stata SE (Stata 18). Continuous variables are envisioned as mean ± standard deviation. Categorical variables are presented with absolute and relative frequencies and are visualized by bar charts. Metric variables are expressed as arithmetic mean and standard deviation or median with corresponding interquartile range. Groupwise comparisons were performed using the student *t*-test, Fisher exact test, Kruskal-Wallis test, and one-way analysis of variance. Additionally, we used correlation and linear regression models to test the association between iECV and ECV and clinical and laboratory baseline variables. For all analyses, the level of significance was set to 0.05.

Survival and freedom from HF hospitalization were displayed using the Kaplan-Meier method and compared using the log-rank test. Correlation and regression models were used to test the association between ECV and iECV and clinical and imaging baseline variables. Kaplan-Meier estimations and Cox regression models were used to investigate the association between ECV, iECV, predefined covariables, and the primary endpoint.

## 3. Results

### 3.1. Demographics

**Figure 1:**
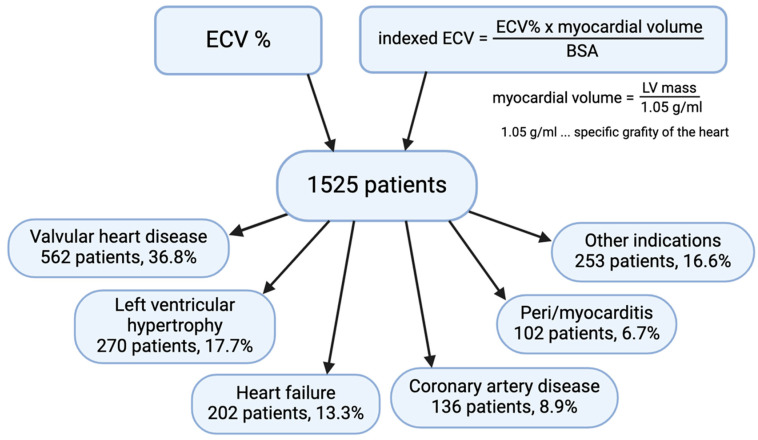
Patient flow and indications for our study cohort.

In total, 1525 consecutive patients were included. The mean age was 65 ± 18 years, and 44% of participants were female. The clinical indications for CMR are shown in Figure 1. The mean conventional ECV was 29 ± 9%, and the mean iECV was 21 ± 13 mL/m^2^.

In this study, we were able to include 1525 patients. ECV was measured and iECV was calculated. Both conventional ECV and iECV provided profound prognostic information about the risk of HF hospitalization and death. However, iECV, which is more complex to determine, provided no additional benefit.

### 3.2. Association Between iECV/ECV and Baseline Parameters

The baseline characteristics, stratified by median iECV (18 mL/m^2^) and ECV (27%), are displayed in Table 1. Overall, iECV was associated with age (R = 0.187, *p* < 0.001) and sex (male, 23.8 mL/m^2^; female, 18.0 mL/m^2^, *p* < 0.001). Patients with an iECV value above the median were more likely to present with atrial fibrillation, hypertension, diabetes mellitus, chronic kidney disease, and previous myocardial infarction. In line with the increased comorbidity burden, these patients had a higher medication intake. Linear and logistic regression, demonstrating the association between iECV and baseline variables, are shown in Table 2.

ECV measures above the median were significantly related to age (R = 0.356, *p* < 0.001) and BSA (R= −4.97, *p* < 0.001). Patients with an ECV value above the median were more likely to present with atrial fibrillation, hypertension, chronic obstructive lung disease, chronic kidney disease, and peripheral artery disease. In line with the increased comorbidity burden, these patients had a higher medication intake. Linear and logistic regression, demonstrating the association between ECV and baseline variables, are shown in Table 3.

### 3.3. Association Between iECV/ECV and Cardiac Magnetic Resonance Parameters

The baseline CMR characteristics, stratified by median iECV (18 mL/m^2^) and ECV (27%), are displayed in Table 4. Patients with iECV and ECV values above the median presented with significantly higher LV mass, poorer LV function, and elevated LV volume (all *p* < 0.001). We also observed poorer right ventricular ejection fraction and higher right ventricular volumes (*p* < 0.001) in patients with iECV and ECV above the median.

### 3.4. Association Between ECV/iECV and Outcome

During follow-up (mean 52 ± 36 months), 414 (27%) events occurred. These included 224 deaths (15%) (89 deaths due to CVD (including eight patients with amyloidosis), 42 patients suffering from cancer, 84 patients with other causes, and 10 patients with infectious diseases). Additionally, 190 hospitalizations for cardiac decompensation (12%) were recorded.

**Figure 2:**
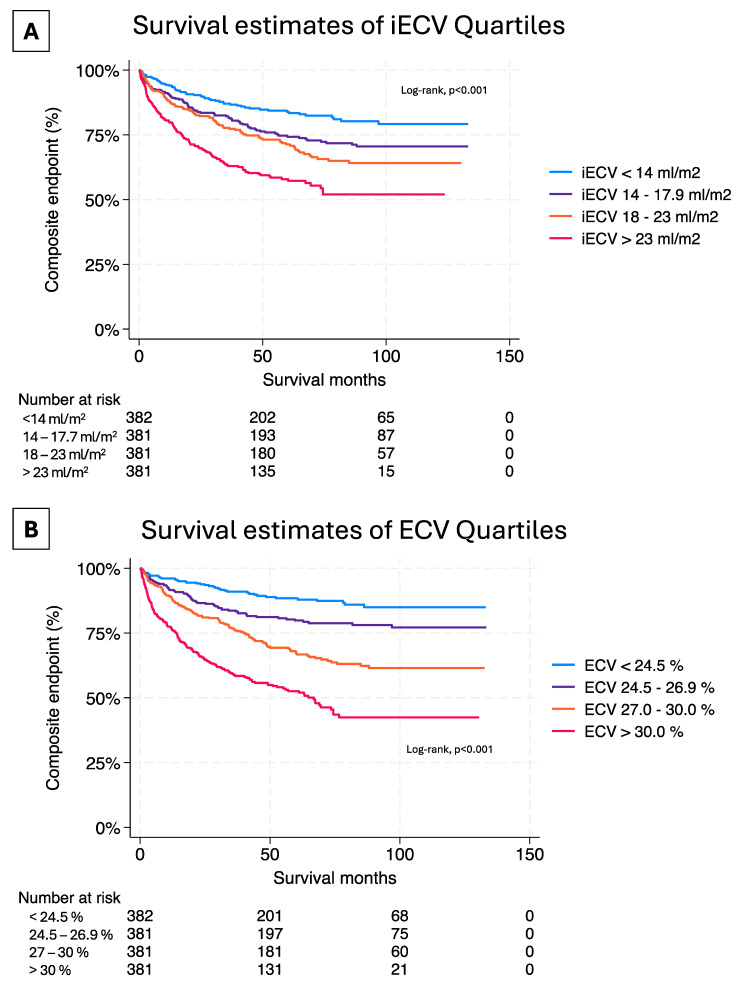
Survival estimates of iECV and ECV quartiles.

**Figure 3:**
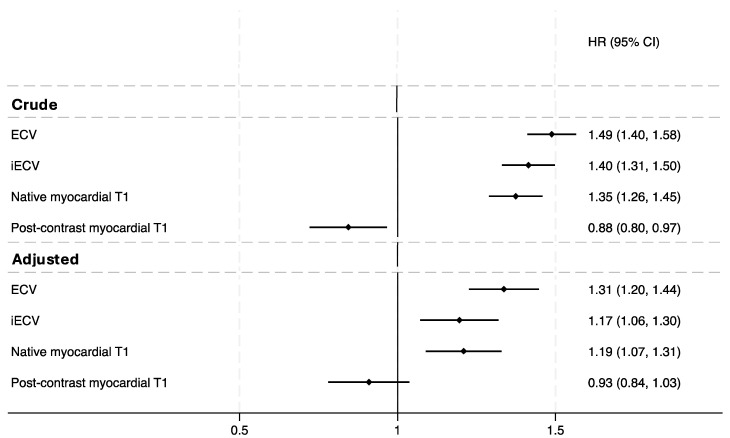
Hazard ratios of ECV, iECV, native myocardial T1 time, and post-contrast myocardial T1 time were adjusted for age, sex, NT-proBNP levels, and ventricular function.

Patients with both ECV and iECV above the median experienced significantly worse outcomes (ECV:HR = 1.03, 95% CI = 1.02–1.05, *p* < 0.001; iECV:HR = 1.01, 95% CI = 1.004–1.02, *p* = 0.002; Figure 2). In a multivariate analysis adjusted for age, sex, ventricular function, and NT-proBNP levels, the conventional ECV remained associated with our composite endpoint (*p* < 0.001). At the same time, iECV demonstrated a slightly less significant association (*p* = 0.002) (seen in the central illustration and in Figure 3). Kaplan-Meier analysis, stratified by conventional ECV tertiles, indicated higher event rates in all quartiles (*p* < 0.001 by log-rank test; see Figure 2B). Similar results were obtained for iECV quartiles (*p* < 0.001 by log-rank test, Figure 2A).

To allow for a head-to-head comparison of ECV and iECV, we report adjusted HR per 1-SD increase of each parameter. We found that conventional ECV demonstrated a higher strength of association with clinical outcomes compared to iECV (ECV:HR = 1.31, 95% CI = 1.20–1.44, *p* < 0.001; iECV:HR = 1.17, 95% CI = 1.06–1.29, *p* = 0.002; central illustration)

### 3.5. Predictive Value of iECV and ECV in Patients with Aortic Valve Disease

#### 3.5.1. Aortic Regurgitation

Overall, 350 patients suffered from chronic AR. Patients with moderate to severe AR presented with significantly lower ECV values compared to the remainder without AR (significant AR: 27.6 ± 5.3% versus no AR: 29.5 ± 9.4%; *p* = 0.002), while iECV did not differ between patients with and without AR (significant AR: 21.34 ± 8.9 ml/m^2^ versus no AR: 21.2 ± 13.8 ml/m^2^; *p* = 0.809).

On age- and sex-adjusted linear regression, both iECV and ECV were significantly associated with AR severity (iECV: *p* = 0.003, ECV: *p* < 0.001).

**Figure 4:**
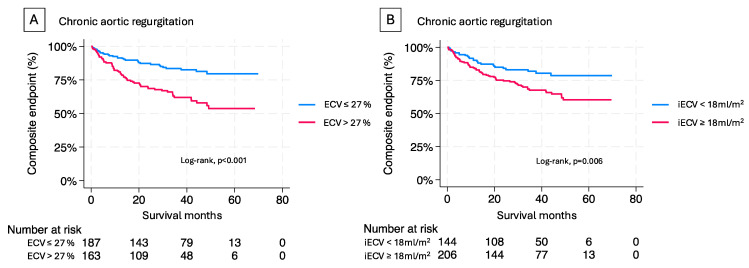
Kaplan-Meier estimates of composite endpoint in patients with aortic regurgitation. Kaplan-Meier curves of patients with chronic aortic regurgitation classified by CMR with (**A**) ECV and (**B**) iECV above and below the median.

Kaplan-Meier analyses of chronic AR patients stratified by median ECV and iECV showed a significantly worse outcome for patients above the median (ECV *p* < 0.001 by log-rank test, and iECV *p* = 0.006 by log-rank test; see Figure 4).

#### 3.5.2. Aortic Stenosis

In 949 patients, the maximum velocity (Vmax) across the aortic valve was above 1.5 m/s (mean Vmax 2.16 ± 1.2 m/s). Furthermore, 133 patients suffered from high-gradient AS (mean Vmax 4.6 m/s), and 43 patients from low-flow, low-gradient AS (mean Vmax 2.8 ± 0.6 m/s). Maximum aortic valve velocity correlated significantly with ECV (R = −0.18, *p* < 0.001) but not iECV (R = −0.03, *p* = 0.40).

**Figure 5:**
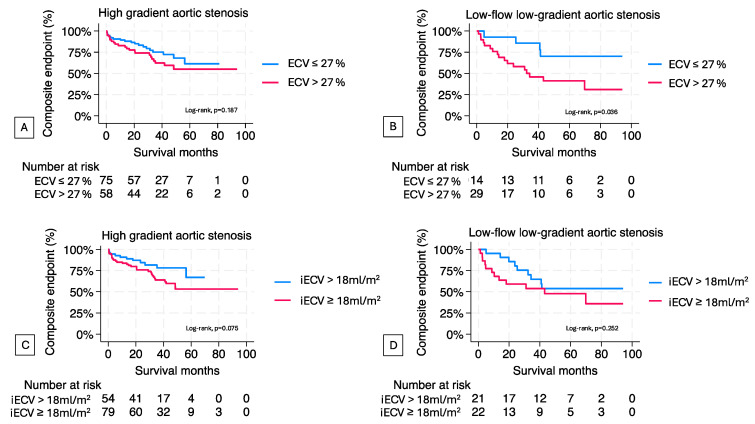
Kaplan-Meier estimates of composite endpoint in patients with aortic stenosis. Kaplan-Meier curves of patients with (**A**) high-gradient aortic stenosis and (**B**) low-flow low-gradient aortic stenosis stratified by median of ECV and in (**C**,**D**) by median of iECV.

Kaplan-Meier analyses of AS patients, stratified by the median values of ECV and iECV, indicated worse outcomes in patients with both iECV and ECV above the median (Figure 5A,C). However, these differences were not statistically significant, except for patients with ECV above the median in low-flow, low-gradient AS, who had a significantly higher risk of adverse outcomes (HR = 3.06, 95% CI = 1.02–9.16, *p* = 0.046) (Figure 5B,D).

## 4. Discussion

Myocardial T1 mapping is a well-established method for assessing myocardial extracellular matrix expansion and has been validated histologically [13,14]. Previous studies have demonstrated that conventional ECV, as measured by CMR T1 mapping, is a reliable predictor of HF hospitalization and mortality [15,16]. ECV effectively differentiates between adaptive and maladaptive hypertrophy, as seen in athletes’ hearts (cellular hypertrophy) versus hypertrophic cardiomyopathy (extracellular matrix expansion) [17,18]. In conditions of chronically elevated pre- or afterload, such as AR or AS, maladaptive remodeling occurs, with extracellular matrix expansion and progressive myocardial fibrosis driving the transition from hypertrophy to HF. This mechanism is well-documented in AS [6,7] and other forms of HF [19,20].

CMR-derived ECV is a potent prognostic biomarker, particularly in aortic valve disease. (2) Additionally, CMR-derived left ventricular (LV) mass is a recognized predictor of outcomes in AS [21,22]. The recently introduced iECV integrates ECV with indexed LV mass into a single measure, hypothesized to better reflect pathological changes in specific conditions.

In 166 patients with AS, iECV showed a significant correlation with histological fibrosis and distinguished patients with AS from healthy controls [7]. Moreover, among these patients, iECV was reported as the only T1 mapping parameter capable of differentiating AS severity grades and was similarly effective in assessing patients with varying grades of aortic insufficiency (AI) [6]. However, these findings were challenged by a study on low-flow, low-gradient AS, which found no differences in ECV or iECV between patients with or without flow reserve during dobutamine stress echocardiography [23].

The utility of iECV has also been evaluated in postoperative settings, particularly after aortic valve replacement [6]. In a study of 99 patients (32 with AR, 67 with AS), iECV decreased postoperatively in AR patients, while ECV remained unchanged, suggesting that iECV may better differentiate between intracellular and extracellular changes. However, the study’s small sample size limits its conclusions.

In another study of 175 AR patients, iECV correlated significantly with regurgitation severity, whereas ECV and replacement fibrosis did not [24]. However, most patients had mild-to-moderate AR, limiting the study’s generalizability. Similarly, a study of 104 patients undergoing surgery for chronic primary mitral regurgitation (MR) found postoperative reductions in both ECV and iECV. Interestingly, higher preoperative iECV was associated with worse postoperative LV function, possibly due to reductions in LV mass [8]. However, these changes were not linked to clinical outcomes.

Outside valve disease, the prognostic value of iECV has been less explored. In a 4-year follow-up of 140 HFpEF patients, iECV was significantly associated with outcomes, even after adjusting for clinical and functional factors [25]. However, ECV was also a strong predictor, and a direct comparison between the two parameters was not performed.

iECV could become the topic in terms of characterizing myocardial tissue on computed tomography (CT). Initial studies have shown that CT could play an important role in the future. CT techniques have already shown a strong correlation and good reliability in ECV quantification compared to CMR. In addition, CT is much more feasible [26].

Overall, existing studies on iECV are limited by small sample sizes, short follow-ups, and narrow patient populations (primarily valve disease or HFpEF), raising questions about its generalizability as a biomarker.

Our study is the first to comprehensively compare iECV and ECV in a broad CMR cohort of 526 patients, including those with aortic valve disease. We found no evidence that iECV offers superior prognostic value compared to ECV in predicting HF hospitalizations or mortality. Both parameters correlated significantly with disease severity in AS and AR, but iECV showed no additional clinical utility. Moreover, its complexity and the time required for calculation reduce its practicality for routine use.

Future research should focus on identifying specific patient populations or scenarios where iECV provides meaningful advantages over traditional ECV, especially in terms of prognostic utility and clinical decision-making. Until then, the additional resources required for iECV calculation must be weighed against its currently limited incremental value.

## 5. Limitations

This study represents the largest investigation to date comparing iECV and ECV for outcome prediction. Despite the large sample size, the single-center design may limit generalizability, although it ensured consistency in CMR acquisition and analysis. While patients with overt cardiac decompensation were excluded, the potential presence of subclinical congestion could have influenced the findings.

## 6. Conclusions

In our large CMR cohort, conventional myocardial ECV provided robust prognostic information regarding HF hospitalization and mortality risk. The newer iECV metric did not offer added prognostic value in either the overall cohort or the subgroup with aortic valve disease. Contrary to prior studies, we did not find evidence supporting the superior predictive strength of iECV for all-cause mortality or HF hospitalizations.

## Figures and Tables

**Table 1:** Baseline characteristics stratified by median iECV and ECV.

	Total	iECV < 18 mL/m^2^	iECV ≥ 18 mL/m^2^	*p*-Value	ECV ≤ 27%	ECV > 27%	*p*-Value
	N = 1525	N = 760	N = 765		N = 762	N = 763	
**Baseline parameters**							
Age (years)	65.5 (17.5)	61.0 (19.0)	70.4 (14.2)	**<0.001**	62.0 (18.8)	69.0 (15.3)	**<0.001**
Female (%)	44.13%	52.24%	36.08%	**<0.001**	42.52%	45.74%	0.21
BSA, m^2^	1.91 (0.23)	1.93 (0.23)	1.90 (0.24)	0.031	1.95 (0.22)	1.88 (0.24)	**<0.001**
**Comorbidities**							
Previous myocardial infarction (%)	26.23%	21.97%	30.46%	**<0.001**	25.98%	26.47%	0.83
Atrial fibrillation (%)	24.10%	20.18%	27.97%	**<0.001**	15.66%	32.50%	**<0.001**
Hypertension (%)	51.74%	48.16%	55.29%	**0.005**	47.11%	56.36%	**<0.001**
Diabetes mellitus (%)	16.00%	12.24%	19.74%	**<0.001**	14.17%	17.82%	0.052
Chronic obstructive lung disease (%)	5.31%	4.61%	6.01%	0.22	3.02%	7.60%	**<0.001**
Hyperlipidemia (%)	29.38%	28.16%	30.59%	0.30	30.05%	28.70%	0.56
PAD (%)	0.92%	0.39%	1.44%	**0.033**	0.13%	1.70%	**0.001**
Chronic kidney disease (%)	9.77%	5.26%	14.25%	**<0.001**	5.12%	14.42%	**<0.001**
**Concomitant medication**							
Betablocker (%)	59.21%	52.21%	67.67%	**<0.001**	51.22%	67.07%	**<0.001**
ACE-Inhibitors (%)	28.99%	25.21%	33.68%	**0.018**	27.83%	30.15%	0.51
Angiotensin-Receptor-Inhibitors (%)	26.51%	22.16%	31.68%	**0.006**	21.41%	31.45%	**0.003**
Calcium-Inhibitors (%)	18.38%	13.89%	23.89%	**0.001**	14.72%	22.02%	**0.016**
T-ASS (%)	34.76%	28.45%	42.52%	**<0.001**	35.17%	34.35%	0.83
Coumadin (%)	13.49%	14.21%	12.59%	0.55	11.11%	15.89%	0.076
NOACS (%)	19.18%	14.44%	24.92%	**<0.001**	12.54%	25.76%	**<0.001**
Diuretics (%)	38.25%	32.69%	45.00%	**0.001**	28.10%	48.35%	**<0.001**
**Laboratory parameters**							
eGFR, mL/min/1.73 m^2^ (MDRD formula)	70.89 (28.89)	76.26 (27.92)	66.62 (28.96)	**<0.001**	76.82 (27.22)	66.29 (29.33)	**<0.001**
Hematocrit, %	39.57 (5.43)	41.00 (4.79)	38.14 (5.64)	**<0.001**	41.61 (4.58)	37.53 (5.45)	**<0.001**
Platelet count, g/L	227.94 (79.89)	233.97 (68.77)	223.09 (87.58)	**0.021**	226.92 (70.11)	228.75 (86.92)	0.70
INR	1.19 (0.42)	1.16 (0.39)	1.21 (0.44)	0.060	1.11 (0.29)	1.25 (0.48)	**<0.001**
Bilirubin mg/dl	0.67 (0.54)	0.64 (0.61)	0.70 (0.48)	0.10	0.62 (0.39)	0.72 (0.63)	**0.001**
Albumin, g/L	41.10 (5.21)	42.27 (4.61)	40.24 (5.46)	**<0.001**	42.62 (4.50)	39.94 (5.42)	**<0.001**
Cholinesterase, U/L	8.49 (16.62)	9.09 (17.31)	8.02 (16.06)	0.30	9.75 (19.51)	7.50 (13.91)	**0.027**
AP, U/L	81.11 (48.09)	76.41 (39.46)	84.89 (53.77)	**0.003**	72.96 (30.11)	87.56 (57.73)	**<0.001**
AST, U/L	29.51 (23.69)	28.13 (18.33)	30.61 (27.19)	0.078	29.13 (28.38)	29.81 (19.26)	0.63
ALT, U/L	28.52 (29.62)	28.27 (22.37)	28.72 (34.30)	0.80	31.10 (37.83)	26.49 (20.82)	**0.009**
gGT, U/L	65.52 (93.27)	52.55 (77.22)	75.69 (103.07)	<0.001	48.41 (71.21)	78.89 (105.54)	**<0.001**
Serum NT-proBNP, pg/mL	2736.16 (5141.42)	1145.47 (2478.89)	3956.56 (6212.88)	<0.001	1317.35 (2853.18)	3866.47 (6177.37)	**<0.001**
Total Cholesterol, mg/dL	163.50 (73.29)	173.24 (95.89)	156.01 (48.07)	<0.001	168.49 (46.02)	159.53 (89.10)	**0.050**
LDL-Cholesterin, mg/dL	88.83 (38.74)	94.14 (39.11)	84.78 (38.00)	<0.001	95.32 (40.02)	83.70 (36.94)	**<0.001**
Leukozyten	7.34 (2.54)	7.36 (2.13)	7.33 (2.82)	0.85	7.40 (2.19)	7.30 (2.78)	0.54
Hämoglobin	12.85 (1.97)	13.34 (1.78)	12.47 (2.03)	**<0.001**	13.62 (1.67)	12.26 (1.98)	**<0.001**
Ferritin, mcg/dL	199.78 (334.39)	199.45 (431.45)	199.99 (256.41)	0.99	232.39 (359.85)	180.54 (318.72)	0.35
CRP, mg/dL	0.98 (1.92)	0.85 (1.65)	1.15 (2.20)	**0.042**	0.87 (1.86)	1.10 (1.97)	0.12

y indicates years; BSA, body surface area; CAD, coronary artery disease; PCI, percutaneous coronary intervention; CABG, coronary artery bypass grafting; MI, myocardial infarction; PAD, periphery artery disease; ACE, angiotensin-converting enzyme; eGFR, estimated glomerular filtration rate; INR, International Normalized Ratio; AP, alcalic phosphate; AST aspartate aminotransferase; ALT alanine aminotransferase; gGT, gamma-glutamyltransferase; NT-proBNP, N-terminal prohormone of brain natriuretic peptide; CRP, C-reactive protein; Bold values indicate *p* ≤ 0.05.

**Table 2:** Linear and logistic regression demonstrating the association between iECV and baseline variables.

iECV	Linear Regression			Age- and Sex-Adjusted		
Parameter	Coeff.	95%Conf-Interval	*p*-Value	Coeff.	95%Conf-Interval	*p*-Value
Age, y (in 10-year increase)	1.37	1.01–1.73	**<0.001**	1.51	1.16–1.86	**<0.001**
BSA, m^2^	−1.06	−3.821–1.71	0.454	−7.89	−10.86–−4.91	**<0.001**
Myocardial native T1, ms	0.13	0.12–0.14	**<0.001**	0.13	0.12–0.14	**<0.001**
Blood native T1, ms	0.01	0.01–0.02	**<0.001**	0.01	0.01–0.02	**<0.001**
Myocardial post T1, ms	−0.09	−0.10–−0.07	**<0.001**	−0.08	−0.09–−0.07	**<0.001**
Blood post T1, ms	0.07	0.06–0.09	**<0.001**	0.07	0.06–0.09	**<0.001**
eGFR, mL/min/1.73 m^2^ (MDRD formula)	−0.07	−0.10–−0.04	**<0.001**	−0.06	−0.09–−0.03	**<0.001**
Hematocrit, %	−0.03	−0.44–−0.21	**<0.001**	−0.32	−0.45–−0.20	**<0.001**
Serum NT-proBNP, pg/mL	0.87	0.73–1.02	<0.001	0.84	0.70–0.98	**<0.001**
HbA1C, %	1.13	0.03–2.24	**0.044**	0.74	−0.35–1.84	0.181
CRP, mg/dL	0.15	−0.33–0.63	0.62	0.04	−0.43–0.50	0.15
LA volume/BSA, mL/m^2^	0.46	0.30–0.62	**<0.001**	0.50	0.34–0.65	**<0.001**
RA volume/BSA, mL/m^2^	0.43	0.24–0.63	**<0.001**	0.37	0.18–0.56	**<0.001**
LVEF, %	−0.24	−0.29–−0.20	**<0.001**	−0.18	−0.23–−0.14	**<0.001**
RVEF, %	−0.26	−0.32–−0.21	**<0.001**	−0.20	−0.26–−0.14	**<0.001**
LVEDV/BSA, mL/m^2^	0.11	0.85–0.13	**<0.001**	0.09	0.07–0.11	**<0.001**
RVEDV/BSA, mL/m^2^	0.09	0.07–0.12	**<0.001**	0.08	0.05–0.10	**<0.001**
IVS, mm	2.22	2.09–2.33	**<0.001**	2.14	2.01–2.27	**<0.001**
CMR-ECV, %	1.23	1.19–1.27	**<0.001**	1.20	1.16–1.24	**<0.001**
	**Logistic Regression**			**Age, and sex-adjusted**		
	**Odds Ratio**	**95%Conf-Interval**	***p*-value**	**Coeff.**	**95%Conf-Interval**	***p*-value**
Sex, male, %	5.65	4.39–6.92	**<0.001**	6.08	4.84–7.33	**<0.001**
Severe aortic regurgitation, %	0.0002	−0.001–0.002	0.809	0.002	−0.004–−0.009	**0.003**
Severe aortic stenosis, %	−1.48	−4.06–1.11	0.262	−2.95	−5.50–−0.40	**0.023**
Hypertension, %	0.61	−0.72–1.93	0.90	−1.29	−2.65–0.06	0.062
Atrial fibrillation, %	2.66	1.16–4.17	**0.001**	1.05	−0.45–2.56	0.170
Diabetes mellitus, %	1.15	−0.60–2.91	0.198	−0.07	−1.77–1.64	0.94
Hyperlipidemia, %	−0.32	−1.73–1.10	0.659	−1.59	−2.97–−0.21	0.024
Chronic obstructive lung disease, %	−0.30	−3.18–2.57	0.836	−1.26	−4.85–1.49	0.368
CAD, %	0.51	−0.96–1.97	0.497	−2.06	−3.52–−0.60	**0.006**
Previous PCI, %	−0.21	−3.13–2.71	0.887	−2.09	−4.95–0.78	0.153
PAD (%)	4.67	−2.08–11.42	0.175	3.19	−3.27–9.65	0.333
Chronic kidney disease (%)	5.81	3.66–7.96	**<0.001**	4.33	2.24–6.41	**<0.001**

CAD indicates coronary artery disease; VHD, valvular heart disease; HF, heart failure; LA, left atrium; BSA, body surface area; RA, right atrium; IVS, intraventricular septum; LV, left ventricular; LVEF, left ventricular ejection fraction; LVSV, left ventricular stroke volume; LVEDV, left ventricular end-diastolic volume; LVESV, left ventricular end-systolic volume; LVCO, left ventricular cardiac output; RVEF, right ventricular ejection fraction; RVSV, right ventricular stroke volume; RVEDV, right ventricular end-diastolic volume; RVESV, right ventricular end-systolic volume; RVCO, right ventricular cardiac output; Bold values indicate *p* ≤ 0.05.

**Table 3:** Linear and logistic regression demonstrating the association between ECV and baseline variables.

ECV	Linear Regression			Age- and Sex-Adjusted		
Parameter	Coef.	95%Conf-Interval	*p*-Value	Coef.	95%Conf-Interval	*p*-Value
Age, y (in 10-year increase)	0.88	0.63–1.13	**<0.001**	0.93	0.68–1.17	**<0.001**
BSA, m^2^	−2.56	−4.44–−0.69	**0.007**	−4.97	−7.05–−2.90	**<0.001**
Myocardial native T1, ms	0.10	0.09–0.10	**<0.001**	0.09	0.07–0.10	**<0.001**
Blood native T1, ms	0.01	0.01–0.02	**<0.001**	0.01	0.01–0.01	**<0.001**
Myocardial post T1, ms	−0.09	−0.10–−0.07	**<0.001**	−0.08	−0.09–−0.07	**<0.001**
Blood post T1, ms	0.07	0.06–0.01	**<0.001**	0.07	0.06–0.09	**<0.001**
eGFR, mL/min/1.73 m^2^ (MDRD formula)	−0.06	−0.07–−0.04	**<0.001**	−0.06	−0.08–−0.04	**<0.001**
Hematocrit, %	−0.27	−0.37–−0.18	**<0.001**	−0.30	−0.40–−0.20	**<0.001**
Serum NT-proBNP, pg/mL	0.53	0.43–0.64	**<0.001**	0.51	0.42–0.62	**<0.001**
HbA1C, %	0.69	−0.07–1.46	0.075	0.57	−0.21–1.34	0.152
CRP, mg/dL	0.22	−0.13–0.56	0.213	0.15	−0.19–0.49	0.384
LA volume/BSA, mL/m^2^	0.30	0.19–0.40	**<0.001**	0.28	0.17–0.39	**<0.001**
RA volume/BSA, mL/m^2^	0.38	0.24–0.52	**<0.001**	0.33	0.19–0.47	**<0.001**
LVEF, %	−0.14	−0.17–−0.11	**<0.001**	−0.12	−0.15–−0.08	**<0.001**
RVEF, %	−0.22	−0.26–−0.18	**<0.001**	−0.19	−0.23–−0.16	**<0.001**
LVEDV/BSA, mL/m^2^	0.14	−0.00–0.03	0.081	0.01	−0.07–0.02	0.285
RVEDV/BSA, mL/m^2^	0.06	0.04–0.07	**<0.001**	0.05	0.04–0.07	**<0.001**
IVS, mm	0.99	0.89–1.09	**<0.001**	0.96	0.85–1.07	**<0.001**
iECV, mL/m^2^	0.57	0.55–0.59	**<0.001**	0.58	0.56–0.60	**<0.001**
	**Logistic Regression**			**Age, and sex-adjusted**		
	**Odds Ratio**	**95%Conf-Interval**	***p*-value**	**Coef.**	**95%Conf-Interval**	***p*-value**
Sex, male, %	1.67	0.80–2.55	**<0.001**	1.94	1.07–2.80	**<0.001**
Severe aortic regurgitation, %	−0.005	−0.007–−0.002	**<0.001**	−0.008	−0.01–−0.006	**<0.001**
Severe aortic stenosis, %	−3.70	−5.41–−2.00	**<0.001**	−4.82	−6.54–−3.10	**<0.001**
Hypertension, %	−0.59	−1.47–0.28	0.284	−1.62	−2.52–−0.72	**<0.001**
Atrial fibrillation, %	3.30	2.28–4.31	**0.001**	2.43	1.39–3.47	**<0.001**
Diabetes mellitus, %	0.25	−0.94–1.44	0.681	−0.52	−1.71–0.66	0.388
Hyperlipidemia, %	−0.98	−1.94–0.17	**0.046**	−1.80	−2.76–−0.84	**<0.001**
Chronic obstructive lung disease, %	0.86	−1.09–2.81	0.388	0.27	−1.64–2.19	0.780
CAD, %	−1.10	−2.10–−0.11	**0.030**	−2.56	−3.58–−1.55	**<0.001**
Previous PCI, %	−0.38	−2.50–1.74	0.726	−1.22	−3.34–0.90	0.258
PAD (%)	3.10	−1.48–7.69	0.185	2.09	−2.41–6.59	0.363
Chronic kidney disease (%)	4.64	3.18–6.09	**<0.001**	3.86	2.41–5.30	**<0.001**

CAD indicates coronary artery disease; VHD, valvular heart disease; HF, heart failure; LA, left atrium; BSA, body surface area; RA, right atrium; IVS, intraventricular septum; LV, left ventricular; LVEF, left ventricular ejection fraction; LVSV, left ventricular stroke volume; LVEDV, left ventricular end-diastolic volume; LVESV, left ventricular end-systolic volume; LVCO, left ventricular cardiac output; RVEF, right ventricular ejection fraction; RVSV, right ventricular stroke volume; RVEDV, right ventricular end-diastolic volume; RVESV, right ventricular end-systolic volume; RVCO, right ventricular cardiac output; Bold values indicate *p* ≤ 0.05.

**Table 4:** Baseline CMR characteristics stratified by median of iECV and ECV.

	Total	iECV **<** 18 mL/m^2^	iECV **≥** 18 mL/m^2^	*p*-Value	ECV **≤** 27%	ECV **>** 27%	*p*-Value
	N = 1525	N = 760	N = 765		N = 762	N = 763	
CMR parameters							
LV-Mass (g)	144.65 (56.32)	104.86 (27.09)	179.51 (52.02)	**<0.001**	137.00 (51.04)	151.81 (60.01)	**<0.001**
LA volume, mL	37.81 (6.85)	36.51 (7.12)	39.08 (6.32)	**<0.001**	36.62 (6.87)	38.98 (6.64)	**<0.001**
LA volume/BSA, mL/m^2^	19.97 (4.15)	19.13 (4.16)	20.80 (3.97)	**<0.001**	18.96 (3.95)	20.97 (4.11)	**<0.001**
RA volume, mL	35.33 (8.64)	34.47 (6.15)	36.44 (10.94)	0.002	34.37 (5.47)	36.33 (10.91)	**0.002**
RA volume/BSA, mL/m^2^	18.44 (4.73)	17.78 (3.26)	19.28 (6.02)	**<0.001**	17.61 (2.92)	19.29 (5.94)	**<0.001**
IVS, mm	12.23 (3.90)	10.44 (2.18)	14.00 (4.40)	**<0.001**	11.69 (3.26)	12.77 (4.39)	**<0.001**
LVEF, %	57.30 (13.75)	60.61 (11.12)	54.07 (15.22)	**<0.001**	60.20 (12.68)	54.37 (14.17)	**<0.001**
LVSV, mL	88.63 (28.77)	86.31 (26.28)	90.93 (30.90)	**0.002**	92.87 (28.36)	84.39 (28.58)	**<0.001**
LVSV/BSA, mL/m^2^	46.25 (13.56)	44.75 (12.24)	47.74 (14.61)	**<0.001**	47.50 (12.69)	45.00 (14.28)	**<0.001**
LVEDV, mL	162.19 (59.89)	146.95 (49.21)	177.31 (65.48)	**<0.001**	160.40 (56.98)	163.98 (62.65)	0.24
LVEDV/BSA, mL/m^2^	84.55 (28.50)	76.04 (22.93)	93.00 (30.89)	**<0.001**	81.98 (26.24)	87.12 (30.39)	**<0.001**
LVESV, mL	73.64 (47.67)	60.71 (33.69)	86.44 (55.43)	**<0.001**	67.46 (42.36)	79.83 (51.74)	**<0.001**
LVESV/BSA, mL/m^2^	38.31 (23.90)	31.31 (16.51)	45.24 (27.76)	**<0.001**	34.43 (20.78)	42.20 (26.09)	**<0.001**
LV-CO, mL	5.73 (3.06)	5.56 (1.82)	5.90 (3.91)	**0.029**	5.92 (1.94)	5.54 (3.86)	**0.015**
LV-CO/BSA, mL/m^2^	3.00 (1.69)	2.88 (0.87)	3.11 (2.21)	**0.009**	3.03 (0.90)	2.96 (2.21)	0.43
RVEF, %	52.78 (11.00)	54.37 (9.86)	51.20 (11.83)	**<0.001**	55.38 (9.78)	50.15 (11.54)	**<0.001**
RVSV, mL	81.39 (26.99)	80.70 (25.34)	82.07 (28.53)	0.32	84.18 (26.21)	78.60 (27.49)	**<0.001**
RVSV/BSA, mL/m^2^	42.44 (12.88)	41.81 (11.90)	43.06 (13.76)	0.059	43.07 (12.00)	41.80 (13.68)	0.055
RVEDV, mL	158.96 (53.84)	152.14 (47.95)	165.70 (58.33)	**<0.001**	154.71 (48.85)	163.20 (58.12)	**0.002**
RVEDV/BSA, mL/m^2^	82.91 (26.02)	78.78 (22.63)	87.00 (28.42)	**<0.001**	79.07 (22.09)	86.75 (28.94)	**<0.001**
RVESV, mL	77.28 (38.92)	71.10 (33.48)	83.40 (42.79)	**<0.001**	70.51 (32.58)	84.07 (43.35)	**<0.001**
RVESV/BSA, mL/m^2^	40.31 (19.63)	36.79 (16.59)	43.78 (21.69)	**<0.001**	35.98 (15.46)	44.65 (22.24)	**<0.001**
RVCO, mL	5.24 (3.24)	5.27 (3.74)	5.21 (2.66)	0.72	5.43 (3.75)	5.06 (2.64)	**0.027**
RVCO/BSA, mL/m^2^	2.73 (1.63)	2.73 (1.85)	2.74 (1.38)	0.88	2.78 (1.85)	2.69 (1.37)	0.29
Myocardial native T1, ms	1023.60 (55.58)	1001.46 (42.81)	1045.59 (58.05)	**<0.001**	999.89 (41.94)	1047.28 (57.43)	**<0.001**
Blood native T1, ms	1613.31 (121.64)	1586.53 (113.43)	1639.93 (123.73)	**<0.001**	1575.25 (107.60)	1651.33 (123.02)	**<0.001**

CAD indicates coronary artery disease; VHD, valvular heart disease; HF, heart failure; LA, left atrium; BSA, body surface area; RA, right atrium; IVS, intraventricular septum; LV, left ventricular; LVEF, left ventricular ejection fraction; LVSV, left ventricular stroke volume; LVEDV, left ventricular end-diastolic volume; LVESV, left ventricular end-systolic volume; LVCO, left ventricular cardiac output; RVEF, right ventricular ejection fraction; RVSV, right ventricular stroke volume; RVEDV, right ventricular end-diastolic volume; RVESV, right ventricular end-systolic volume; RVCO, right ventricular cardiac output; Bold values indicate *p* ≤ 0.05.

## Data Availability

The data presented in this study are available on request from the corresponding author.
